# Improving Fabrication and Performance of Porous Silicon Electron Emission Devices via Functional Layer Resistivity Modulation

**DOI:** 10.3390/nano16050337

**Published:** 2026-03-09

**Authors:** Jinxin Dong, Xiaojing Huyan, Fangzhou Luo, Guanyang Zhang, Qiang Liu, Yawen Li, Tianbao Hu, Yongxun Liu, Shinan Wang, Wenjie Yu

**Affiliations:** 1 School of Microelectronics, Shanghai University, Shanghai 201800, China; 2 Shanghai Institute of IC Materials Co., Ltd., Shanghai 201800, China; 3 Shanghai Industrial μTechnology Research Institute, Shanghai 201800, China; 4 State Key Laboratory of Materials for Integrated Circuits, Shanghai Institute of Microsystem and Information Technology, Chinese Academy of Sciences, Shanghai 200050, China

**Keywords:** porous silicon, electron emission device, resistivity modulation, epi film, ion implantation

## Abstract

To improve the process controllability and fabrication uniformity of porous silicon (PS)-based electron emission devices (EEDs), we employed an epitaxial (epi) silicon film as the functional layer, leveraging its advantages of high crystalline quality and flexibility of resistivity modulation regardless of the substrate. Precise modulation of the epi film resistivity was achieved via ion implantation. We investigated the effects of resistivity modulation on the fabrication process and device performance. This scheme enabled the formation of PS through electrochemical etching without illumination, and therefore etch self-termination. As a direct result, the etching uniformity in both the vertical and horizontal directions is enhanced. It then facilitated the optimization of the oxidation of the PS surface, which is essential for EED performance. The devices exhibited a maximum electron emission current density (*J_e_*) of 80 μA/cm^2^ with high stability. Driven under DC mode at a bias voltage (*V_ps_*) of 23 V, *J_e_* decreased temporarily to 28 μA/cm^2^ after 4 h of continuous operation. This study provides a new feasible approach for research on PS EEDs.

## 1. Introduction

The rapid development of vacuum microelectronic devices imposes stringent requirements on high-performance EEDs. Cutting-edge fields such as electron beam lithography systems [1], field emission flat-panel displays [2], discharge-free gas excitation light sources [3], and micro vacuum sensors [4] are in urgent need of EEDs with a low operating voltage, excellent voltage endurance, and a long lifetime. In 1993, Smith et al. first demonstrated PS field emission array devices, exhibiting their feasibility as cold cathode EEDs [5]. Over the past three decades, PS-based EEDs have emerged as a research hotspot due to their compatibility with conventional CMOS process, low fabrication cost, and ballistic electron transport characteristics [1,6,7,8,9,10,11,12,13,14,15,16]. Between 1995 and 2011, Koshida et al. progressively developed an electron emission model for PS, explicating that electrons injected from the single-crystalline silicon substrate can be stepwise accelerated while quasi-ballistically tunneling through interconnected nanocrystalline silicon (nc-Si) chains formed within the PS layer, and ultimately tunnel through the top electrode to form a directional emission current [6,10,11,12,13]. These continuously refined models have laid the core theoretical foundation for PS-based EEDs. In 1999, Komoda et al. passivated interface defects of PS via rapid thermal oxidation (RTO), realizing the integration of porous polycrystalline silicon EEDs on glass substrates with an increased *J_e_* of 100 μA/cm^2^ [8]. This provided key support for the development of ballistic electron surface-emitting displays (BSDs). In 2000, Koshida et al. formed an oxide film on an nc-Si grain surface via electrochemical oxidation (ECO), revealing the advantages of the low-temperature oxide film growth and dangling bond passivation [9]. In the past decade, He et al. carried out more in-depth study on the optimization of the process of PS EEDs and their application in novel flat electron emission lamps (FEELs). In terms of optimization of the process, He et al. proposed a method combining cathode reduction (CR) treatment and ECO to enhance the electron emission performance of EEDs with emission current densities and efficiencies of 62 μA/cm^2^ and 12.10% [16]. Then they studied the combined effects of the anodization current density and time on the PS morphology and the emission performance, and found the effective anodization current density and anodic electrical charge [17]. Subsequently, they proposed a fabrication process of multilayered porous silicon (MPS)-based devices that is different from that of conventional single-layered porous silicon-based devices. For MPS EEDs, they also studied the effects of electrochemical etching [18], CR [19] and ECO [20] parameters on device performance, and further optimized the emission current density, emission efficiency and emission current fluctuation. In terms of application in FEELs, compared with the conventional fluorescent lamp, He et al. demonstrated a novel FEEL using the PS-based electron emitter as the cold cathode. This kind of FEEL is characterized by a low vacuum requirement, convenient fabrication, favorable energy conversion efficiency and power consumption, giving it good development and application prospects [21,22].

From single-crystalline silicon-based porous structures [5,6,7,9,10,11,16,17,18,19,20,21,22] to polycrystalline silicon-based nanocrystal arrays [1,8,12,13,14,15], significant progress has been made in the electron emission mechanism, structural design, and process optimization of PS-based EEDs. However, issues such as high junction currents, insufficient voltage endurance, and limited device lifetime still hinder large-scale application in high-end vacuum microelectronic fields [23].

Despite considerable progress, existing single-crystalline and polycrystalline silicon-based devices still exhibit limitations, which are closely related to their crystalline structures and electrical characteristics. On one hand, for single-crystalline substrate-based EEDs, previous studies typically employed heavily doped single-crystalline silicon substrates, with a resistivity range of 0.001–0.01 Ω·cm, for several reasons:

1. Low-resistivity substrates can supply sufficient electrons for emission;
2. They have low on-resistance, ensuring that the external bias voltage mainly acts on the PS layer to sufficiently accelerate electrons;
3. Their electrochemical etching process has better controllability [24,25].

However, this kind of EED is prone to excessively high junction currents, leading to Joule heat accumulation. This can cause the collapse of nc-Si chain structures, thereby affecting device operating stability and lifetime [26]. On the other hand, for polycrystalline silicon-based EEDs, obtaining low-resistivity polycrystalline silicon films with the desired quality remains challenging. A notable characteristic of polycrystalline films is their non-uniform grain size distribution and irregular grain boundaries, which impair the controllability of both the fabrication process and the device performance. For example, during electrochemical etching, the etching solution rapidly penetrates the polycrystalline silicon film along the grain boundaries due to the high density of defects therein, and reacts with the underlying single-crystalline silicon substrate. This ultimately forms large etching pits in the substrate, significantly impairing device performance [12]. Additionally, under external bias voltage, defect sites at grain boundaries are more likely to trap carriers, leading to electric field concentration. This can easily form leakage channels and degrade EED electrical performance.

In this new attempt, we demonstrated an EED using an epitaxial (epi) silicon film as the functional layer for PS formation. Compared with conventional single-crystalline and polycrystalline silicon approaches, the key advantages of epi films include high crystalline quality and flexible resistivity modulation regardless of the substrate. We developed a flexible and controllable fabrication process involving ion implantation, electrochemical etching, oxidation, and electrode formation. The evaluation of EED emission performance confirmed that this method yields EEDs with excellent uniformity and stability.

## 2. Fabrication

### 2.1. Fabrication of PS-Based EED on Epitaxial Silicon Film

**Figure 1:**
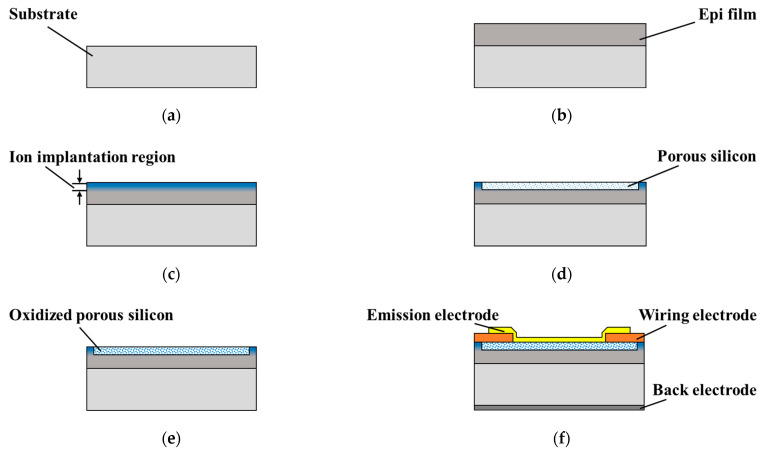
Device fabrication process flow: (**a**) substrate preparation; (**b**) epi film growth by CVD; (**c**) ion implantation and annealing; (**d**) porous silicon formation by anodization; (**e**) oxide film formation by ECO and RTO; (**f**) electrode formation.

**Figure 2:**
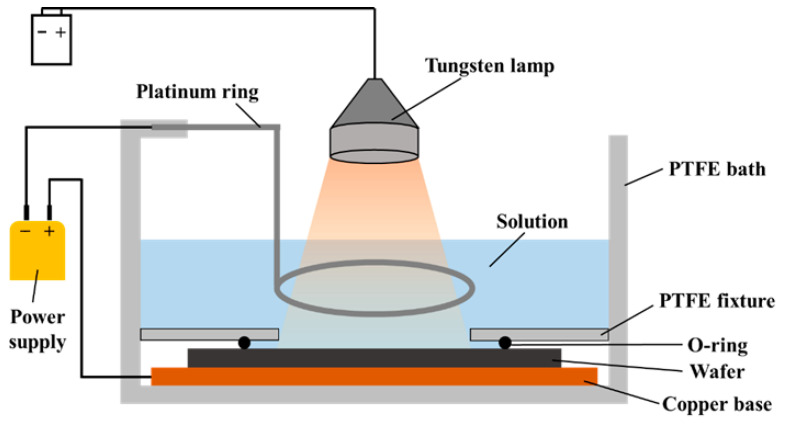
Schematic of electrochemical etching and ECO setup.

The fabrication process is illustrated in Figure 1. As shown in Figure 1a, 8 inch (100) n-type silicon wafers with a resistivity of 0.01–0.05 Ω·cm and a thickness of 625 μm were used as the starting material. The wafers were first cleaned using the standard RCA method [27]. In Figure 1b, a 2 μm thick single-crystalline silicon film was epitaxially grown on each of the wafers via chemical vapor deposition (CVD) at 1100 °C, with SiHCl_3_ gas used as the precursor. The depth-dependent resistivity distribution of the epi film was measured via spreading resistance profiling (SRP), which is shown in Section 3.1. After epi film formation, the wafers were diced into 20 × 20 mm^2^ chips to act as substrates in the following processes. In Figure 1c, phosphorus ion implantation was performed on the epi film at 50 keV with a dose of 2 × 10^15^ cm^−2^. Subsequently annealing was conducted at 900 °C for 30 min in an N_2_ atmosphere to activate the implanted phosphorus atoms and repair lattice damage caused by high-speed ion bombardment. This process formed an equivalent “low-resistivity single-crystalline silicon” region in the top region of the 2 μm epi film while the resistivity in the bottom region remained unchanged. In Figure 1d, a PS structure was formed in the epi film via the electrochemical etching (also called anodization) method in a 1:1 solution of HF (49%):C_2_H_5_OH. The electrochemical etching setup was self-built, with its schematic shown in Figure 2. A pulse current with a duty cycle of 2 ms:8 ms was used to enhance the vertical uniformity of the PS [28]. For as-grown epi films, front-side illumination with a tungsten lamp was necessary for PS formation as the etching rate was found to be extremely low without illumination. However, for the epi films with ion implantation, it was not necessary. In Figure 1e, thin silicon oxide films were formed on the surfaces of the nc-Si grains as well as the PS walls via a combined oxidation method involving ECO and RTO [29]. First, ECO was performed in a 1 mol/L H_2_SO_4_ solution using the setup shown in Figure 2 (without front-side illumination). ECO is known to passivate dangling bonds, Si-H and Si-H_2_ bonds within the PS [12], which are detrimental to EED performance. To improve the dielectric properties of the oxide film, following prior research [12], the sample was annealed at 550 °C for 30 min in a forming-gas (5% H_2_ + 95% N_2_) atmosphere after ECO. To further enhance the structural integrity and uniformity of the oxide film [18], RTO was subsequently performed at 900 °C for 30 min in an O_2_ atmosphere. The final step was electrode formation, as shown in Figure 1f. For the top electrodes, two rounds of lift-off process were performed, including photolithography, physical vapor deposition (PVD) of metals, and removal of photoresists via C_5_H_9_NO dissolution. This resulted in the formation of a wiring electrode (10 nm Ti + 200 nm Au) and an emission electrode (1 nm Ti + 10 nm Au) on the PS surface, wherein the Ti film served as an adhesion layer. For back electrode formation, a 300-nm thick Al layer was sputtered on the backside of the substrate after removing the oxide film grown there during the RTO process.

### 2.2. Performance Testing of EEDs

**Figure 3:**
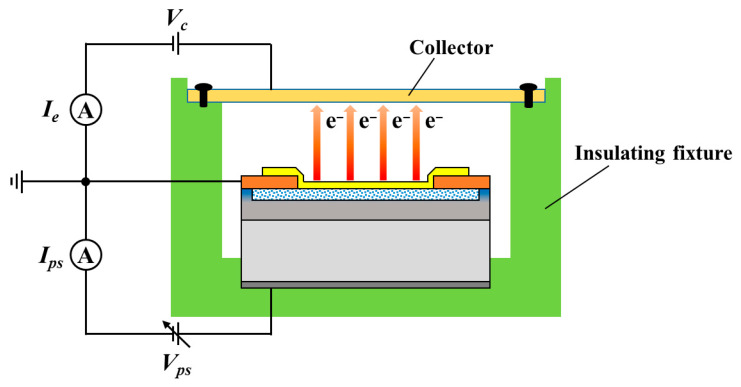
Schematic of the emission testing setup.

Performance testing was conducted in a vacuum environment under ~3 × 10^−4^ Pa. A schematic of the testing setup is shown in Figure 3. An external positive bias voltage *V_ps_* was applied from the top wiring electrode to the back electrode. A collector electrode, positioned approximately 2 mm above the emission electrode, was applied a collector voltage *V_c_* = 200 V relative to the grounded emission electrode. We monitored the diode current *I_ps_* and emission current *I_e_*. The diode current density *J_ps_* and emission current density *J_e_* are calculated according to the junction area *S_ps_* (1.74 × 10^−2^, 2 × 10^−2^, 2.16 × 10^−2^, 2.3 × 10^−2^ cm^−2^, respectively) and the emission area *S_e_* (7.85 × 10^−3^ cm^−2^), respectively. Under the external bias voltage *V_ps_*, electrons are injected from the substrate into the PS region and continuously accelerated while quasi-ballistically passing through the cascaded nc-Si grains and tunneling through the thin oxide films enveloping the grains. The electrons with energy exceeding the work function of the Au emission electrode (~5.1 eV) can tunnel into the vacuum and reach the overhanging collector under *V_c_* [6]. In this study, the emission currents were observed at a bias voltage *V_ps_* much higher than 5.1 eV, and the underlying reason is discussed in Section 3.1. Here, we focus on the variation in the electron emission current density *J_e_* versus the external bias voltage *V_ps_*. Additionally, the time-dependent stability of *J_e_* under a constant *V_ps_* was evaluated.

## 3. Results and Discussion

### 3.1. Effects of Resistivity Modulation on the Fabrication Process

**Figure 4:**
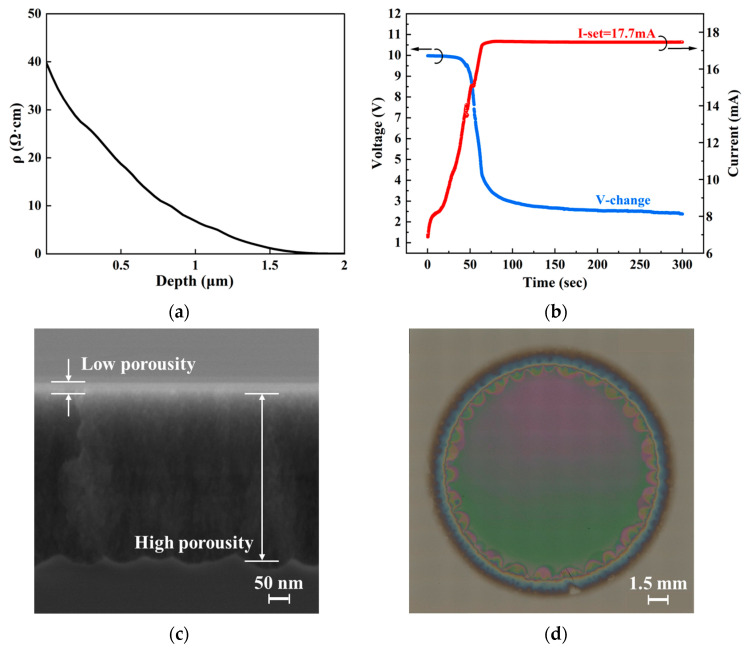
Evaluation of an as-grown epi film and the PS formed therein. (**a**) Resistivity distribution versus film depth. (**b**) Current and voltage variation during constant-current mode electrochemical etching. (**c**) SEM cross section and (**d**) overall plan view (automatically stitched optical microscope images) of the PS.

**Figure 5:**
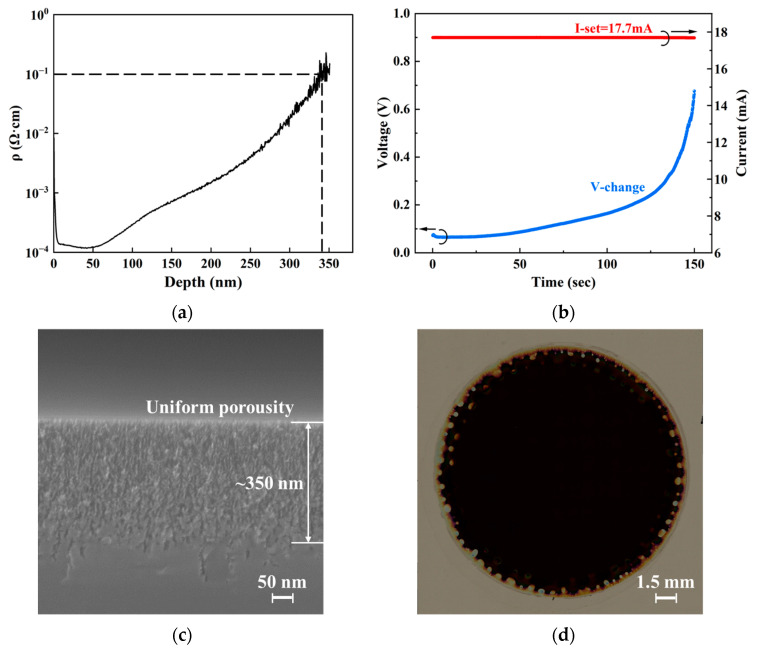
Evaluation of the ion-implanted epi films and the PS formed therein. (**a**) Resistivity distribution versus film depth. (**b**) Current and voltage variation during the constant-current mode electrochemical etching. (**c**) SEM cross section and (**d**) overall plan view (automatically stitched optical microscope images) of the PS.

We studied the effects of resistivity modulation on the fabrication process by comparing as-grown epi films, ion-implanted epi films, and the corresponding PS structures. The results for as-grown epi films are presented in Figure 4, while those for ion-implanted films are shown in Figure 5.

In Figure 4, the depth distribution of the resistivity of the 2 μm thick as-grown epi films was first measured via the SRP method and is shown in Figure 4a. The epi film exhibits a gradient resistivity distribution where the resistivity is the lowest at the film–substrate interface (~0.02 Ω·cm), and the highest at the film surface (~40 Ω·cm). This is the result of impurity diffusion from the heavily doped substrate during the high-temperature epitaxial growth. Figure 4b presents the current and voltage variation during PS formation via electrochemical etching under the galvanostatic mode. In the initial stage, the current failed to reach the set value (17.7 mA, corresponding to a current density of 10 mA/cm^2^) but kept increasing for a considerable period of time (~75 s). The etching current reached the set value and afterwards remained almost constant. We explain this etching current behavior as follows. In the initial stage, the high resistivity at the epi film surface induces a high surface barrier at the etchant–film interface, hindering carrier transport and preventing holes from promptly reaching the surface to participate in the reaction [24]. As the etching proceeds, the continuous decrease in the film resistivity as the etching depth increases results in a reduction in the interface barrier and finally makes it easier for holes to migrate to the interface. Thus, stable electrochemical etching becomes possible under a constant current. The cross-sectional scanning electron microscope (SEM) image of the PS is shown in Figure 4c. The thickness of the PS structure is approximately 500 nm. Vertically, the PS is not uniform and exhibits two regions. The porosity of the PS is low in the top region. This corresponds to the low current density in the initial etching stage. In contrast, the porosity in the underlying region is higher, corresponding to the constant current etching stage. This is unfavorable for the subsequent oxidation processes where uniform penetration of H_2_SO_4_ liquid and O_2_ gas into the bottom of the pores is essential to achieve uniform oxidation in the vertical direction [18]. Furthermore, in the electrochemical etching of a high-resistivity film or substrate, due to the shortage of the hole supply in a dark environment, illumination is typically necessary to excite electron–hole pairs. However, as shown in the typical example in Figure 4d, PS structures formed under illumination in the high-resistivity films exhibited a multicolored surface morphology, indicating notable in-plane non-uniformity. In Figure 4d, semicircular patterns observed along the edge of the etched area were attributed to irregular etching adjacent to the sample-fixing O-ring (see Figure 2), where bubbles accumulated during the etching. The inner diameter of the O-ring is about 15 mm. This PS structure’s non-uniformity was found to lead to non-uniformity in the subsequent oxidation processes, and therefore large device performance variations across the chip.

In Figure 5, the results for ion-implanted films are presented. After ion implantation and annealing in the as-grown epi films, the impurity distribution was measured by secondary ion mass spectrometry (SIMS). Assuming that the implanted impurities were fully activated, the corresponding resistivity distribution was calculated using the SIMS data and are plotted in Figure 5a. It can be seen that the resistivity near the surface of the epi film decreased to approximately 0.001 Ω·cm. The resistivity gradually increased with depth, and reached a value of about 0.1 Ω·cm at a depth of ~350 nm. Figure 5b shows the current and voltage variation during the electrochemical etching where illumination was not used. In Figure 5b, the initial stage of the electrochemical etching corresponds to the low-resistivity region. Due to resistivity modulation, the surface barrier is reduced and holes can easily be transported to the etchant–silicon interface without illumination so that PS formation becomes possible [24]. Thus, the holes are available to participate in the reaction from the starting point of the electrochemical etching, and the entire reaction process can operate under a constant current. This means an almost constant etching rate, resulting in a PS structure that has uniform pore sizes in the vertical direction, as confirmed by the SEM observations shown in Figure 5c. The horizontal distribution uniformity of the PS is also improved. As shown in Figure 5d, no obvious color differences are observed in the PS surface, excluding the irregularly etched edge region adjacent to the O-ring. Additionally, when electrochemical etching proceeds to a certain depth, the resistivity gradually increases due to the corresponding reduction in carriers participating in the reaction, leading to self-termination of the reaction at a specific depth. In Figure 5c, a PS structure with a depth of approximately 350 nm was obtained. This depth corresponds to the depth where the film resistivity increased to about 0.1 Ω·cm in Figure 5a. Even though the etching time was extended, the PS depth did not notably increase. This phenomenon is consistent with Lehmann’s report: the critical resistivity for n-type silicon electrochemical etching without illumination is about 0.1 Ω·cm (corresponding a doping density of about 10^18^ cm^−3^) [24]. Furthermore, the research results of He et al. revealed that the pore sizes of PS are closely related to the electrochemical etching current and time [16,18,30,31]. As demonstrated above, the PS thickness can be precisely controlled by the self-termination effect occurring at the interface between high-resistivity and low-resistivity regions. Therefore, leveraging our resistivity modulation method, it is easy to fabricate PS structures with different pore sizes but almost the same depth simply by controlling the etching conditions, thereby meeting different application requirements. In our study, we controlled the low resistivity in near-surface region and the high resistivity in the bottom region remained unchanged. The high resistivity in the bottom region has three functions:

1. To realize self-termination of the electrochemical etching so that the thickness of the PS structure is vertically uniform and controllable;
2. To prevent the etching from reaching the substrate so that etching pits can be avoided at the film–substrate interface;
3. To restrict the junction current of the device so that Joule heat accumulation is reduced and both the emission efficiency and stability can be improved.

### 3.2. Effect of Resistivity Modulation on Device Performance

**Figure 6:**
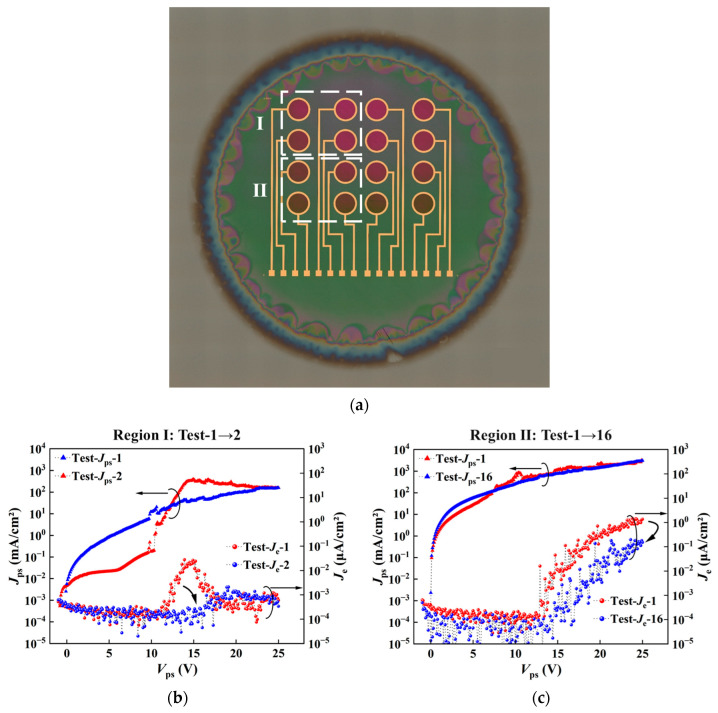
Evaluation of the devices formed in different regions of the chip without ion implantation. (**a**) Overall plan view (automatically stitched optical microscope images) of the devices formed on the chip corresponding to Figure 4d. (**b**,**c**) Emission performance of devices in regions I and II, respectively. Test-(*J_ps_* or *J_e_*)-n indicates the result after n consecutive measurements (here, n = 1, 2 or 16).

**Figure 7:**
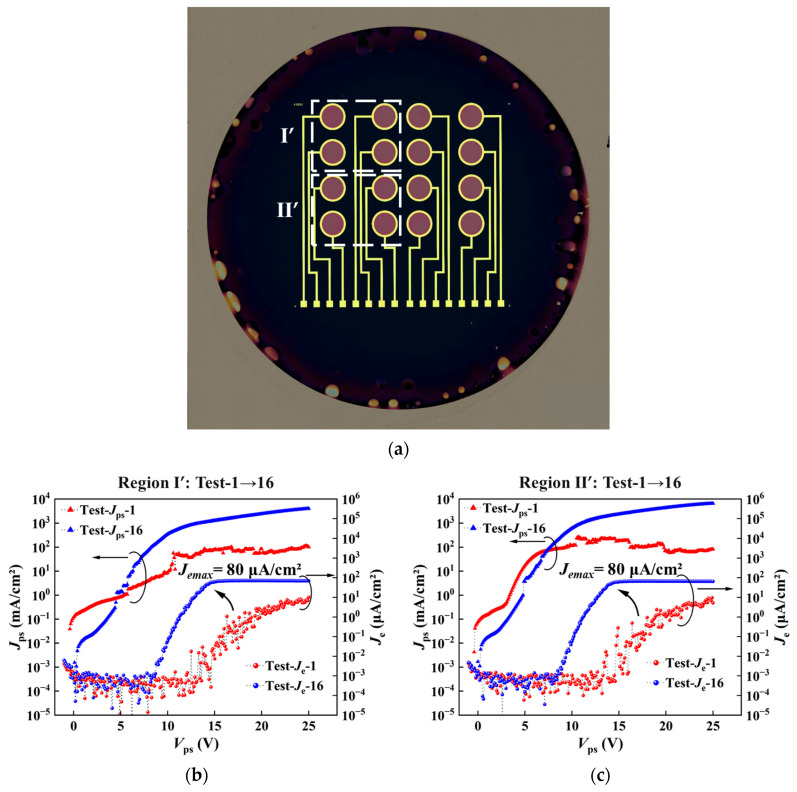
Evaluation of the devices formed in different regions of the chip with ion implantation. (**a**) Overall plan view (automatically stitched optical microscope images) of the devices formed on the chip corresponding to Figure 5d. (**b**,**c**) Emission performance of devices in regions I’ and II’, respectively. Test-(*J_ps_* or *J_e_*)-n indicates the result after n consecutive measurements (here, n = 1 or 16).

We studied the effects of resistivity modulation on the device performance by comparing the electron emissions in devices based on the as-grown and ion-implanted epi films, as shown in Figure 6 and Figure 7, respectively.

First, Figure 6 shows the emission characteristics of devices formed in different regions of the same as-grown epi film. Figure 6a shows an overall plan view of the chip with electrodes formed on the PS surface corresponding to Figure 4d. The emission characteristic of one device in region I is shown in Figure 6b, and that of a device in region II is shown in Figure 6c. In each region, the four devices exhibited similar emission characteristics. For simplicity, only the emission characteristic of one device is presented for each region. Obviously, the *J_e_* values are low with large fluctuations, and differ significantly from each other. As shown in Figure 6b, the *J_e_* of the device in region I decays rapidly when the *V_ps_* reaches approximately 15 V in the first test, and becomes extremely low in the second test. In contrast, the *J_e_* of the device in region II performs a little better, as shown in Figure 6c: the *J_e_* is slightly higher in the first test, and decreases by approximately one order of magnitude after 16 consecutive tests. The poor emission characteristics suggest poor PS structure quality. The differences in *J_e_* between different devices may be attributed to the non-uniformity of the PS structures in different regions, which is obvious from the multicolored appearance of the chip. The non-uniformity of the PS structures may also lead to non-uniform oxidation in different regions. Horizontal non-uniformity causes differences in the acceleration of electrons through the oxide films in different emission regions, and the continuity of the cascaded nc-Si grain emission channels also varies. Ultimately, differences in the quantity and energy of electrons received by the upper collector are reflected in the *J_e_*-*V_ps_* curves.

Second, Figure 7 shows the emission characteristics of devices formed in different regions of the same ion-implanted epi film. Figure 7a shows an overall plan view of the chip with electrodes formed on the PS structure corresponding to Figure 5d. The emission current density of one device in region I’ is shown in Figure 7b, and that of a device in region II’ is shown in Figure 7c. Similar to Figure 6, the four devices in each region exhibited similar emission characteristics so only the emission characteristic of one device is presented for each region for simplicity. However, the emission performance of any device in Figure 7 is greatly improved compared with that in Figure 6. As seen in Figure 7, the emission characteristics of devices from regions I’ and II’ resemble each other, and are quite different from those in Figure 6. In Figure 7, the initial *J_e_* of a device is still low in the first test. But, after 16 consecutive tests, the *J_e_* increases by 1–2 orders of magnitude with significantly reduced fluctuations. The change in *J_e_* after a certain number of *V*_ps_ scans could be attributed to the electroforming effect [31]. Stable electron transport channels gradually form and the electron emission then becomes relatively stable via electroforming. The *J_e_* in Figure 7b and c reaches approximately 80 μA/cm^2^ at an operating voltage of about 25 V and almost saturates thereafter.

One can see that the two devices in different regions exhibit a close resemblance in emission characteristics, which are incomparably superior to those in Figure 6. This is attributed to the better vertical and horizontal uniformity of the electrochemical etching of the sample after ion implantation, which leads to a more uniform distribution of PS and better continuity of cascaded nc-Si grains. Consequently, the uniformity of the subsequent oxidation processes is improved, greatly enhancing the overall uniformity of device emission performance.

It is notable that the threshold voltage in the above devices is quite high, exceeding 10 V. This may not be the intrinsic limitation of the epi film-based EEDs. By further optimizing the total thickness and the resistivity modulation depth in the epi film, the threshold voltage is expected to be lowered to values close to those in single-crystalline substrates and polycrystalline films. In the meantime, the emission performance of EEDs may also be improved.

**Figure 8:**
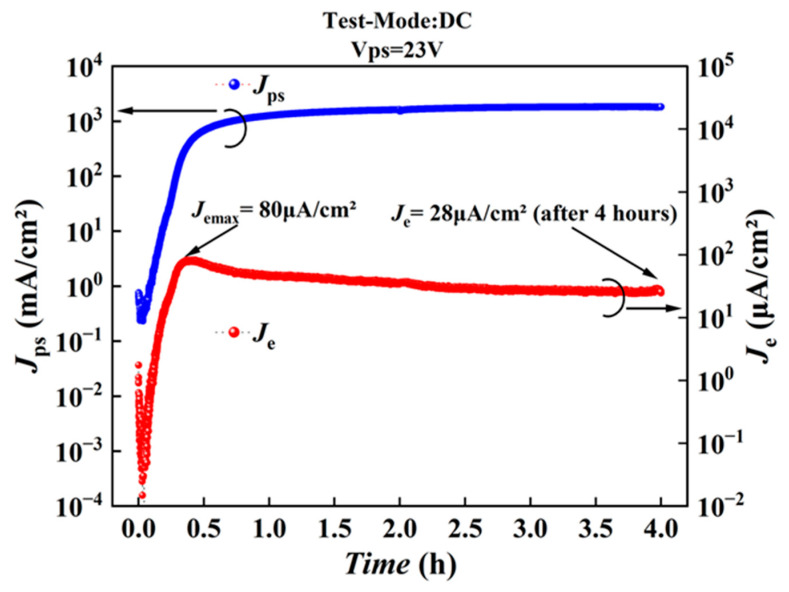
Stability test result of *J_e_* in a device made using the epi film with ion implantation.

Figure 8 shows the result of the emission stability test of a device formed in the ion-implanted epi film. The test was conducted at room temperature under a direct current (DC) mode with *V_ps_* = 23 V, under a vacuum of approximately 3 × 10^−4^ Pa. The sampling period in Figure 8 is 1 s. During the initial stage, the emission current density *J_e_* changed greatly, implying that the device experienced the electroforming process, which took as long as about 0.4 h for this device. Then, the *J_e_* reached a maximum value of about 80 μA/cm^2^, followed by a gradual decrease to about 28 μA/cm^2^ after 4 h of continuous operation.

**Figure 9:**
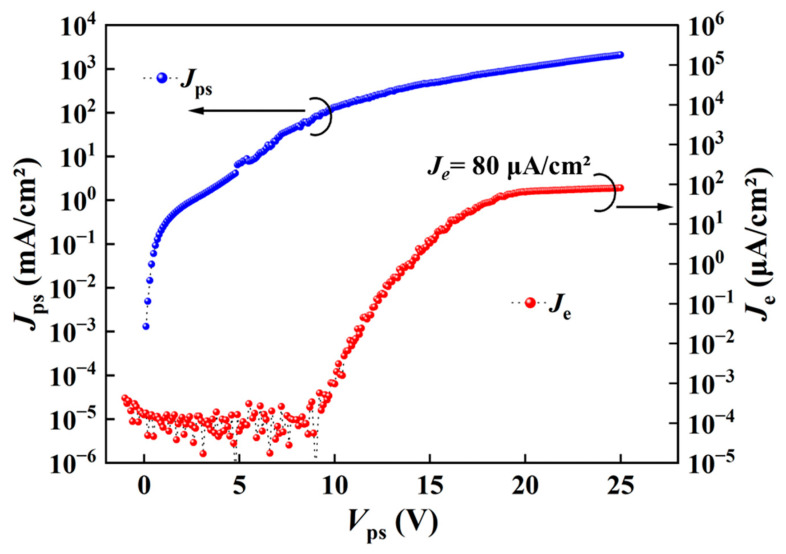
Emission performance (*J_e_* versus *V_ps_*) test result after stability test corresponding to Figure 8.

Figure 9 shows the emission performance of the device after the 4 h stability test in Figure 8. Surprisingly, the device exhibits almost the same emission characteristic as those that had not been subjected to the stability test. Under a *V_ps_* of 25 V, the *J_e_* recovers to the maximum value of 80 μA/cm^2^, as shown in Figure 8. We can then conclude that the device has not degraded significantly even after the 4 h long continuous DC test. The decrease in *J_e_* in Figure 8 does not mean a real decay of the device, but may be caused by certain temporary changes associated with Joule heat accumulation. These results suggest that by adopting optimized driving modes, not the continuous DC mode, superior emission stability can be achieved in such a PS-based EED.

By comparing the device performance of EEDs based on epi films, it is clear that resistivity modulation not only greatly improves the process’s controllability and uniformity, but also the emission characteristics. We have shown above that ion implantation is an effective method for resistivity modulation in high-resistivity films. Alternative methods such as in situ doping during epitaxial growth may work too. For example, in a silicon film epitaxy on a low-resistivity silicon substrate, one can first perform epitaxial growth without impurity doping to obtain a high-resistivity silicon sublayer. Then, in situ impurity doping can be carried out during the remaining epitaxial growth to obtain a lower-resistivity silicon sublayer. Of course, the impurity inter-diffusion during the high-temperature epitaxial growth must be taken into account so that one can obtain a single-crystalline silicon layer with accurate resistivity modulation. The controlled in situ doping method is particularly practical for obtaining a thick low-resistivity silicon sublayer, which is difficult to achieve with ion implantation due to the necessary high implantation energy. Forming PS of an appropriate thickness may help enhance the emission current density of the devices [16,18,28,30,31].

## 4. Conclusions

The feasibility of improving the process controllability and fabrication uniformity of porous silicon-based electron emission devices has been verified via resistivity modulation in an epitaxial film with inherently high resistivity: the film resistivity is reduced in the top region but retained in the bottom region. The low-resistivity top layer ensures uniformity of the porous silicon structure, while the high-resistivity bottom layer enables the electrochemical etching to stop within it and prevents the substrate from abnormal corrosion. The thus-obtained highly uniform porous silicon structure facilitates the growth of high-quality oxide films on its surface, which is critical for device performance. Meanwhile, the high-resistivity bottom layer also functions to regulate the junction current, effectively avoiding the accumulation of destructive Joule heat. The resultant devices exhibit favorable consistency and stability. It is expected that device performance can be enhanced by further optimizing the epi film thickness and resistivity modulation depth.

## Data Availability

The original contributions presented in this study are included in the article. Further inquiries can be directed to the corresponding author(s).
